# Face off: a metabolic enzyme becomes a protein phosphatase

**DOI:** 10.1093/procel/pwad006

**Published:** 2023-02-15

**Authors:** Gaoxiang Zhao, Qian Lin, Zhaoyuan Meng, Xinlei Sheng, Leina Ma, Yingming Zhao

**Affiliations:** Department of Oncology, Qingdao Cancer Institute, The Affiliated Hospital of Qingdao University, Qingdao University, Qingdao 266071, China; Department of Oncology, Qingdao Cancer Institute, The Affiliated Hospital of Qingdao University, Qingdao University, Qingdao 266071, China; Department of Oncology, Qingdao Cancer Institute, The Affiliated Hospital of Qingdao University, Qingdao University, Qingdao 266071, China; Ben May Department for Cancer Research, The University of Chicago, Chicago, IL 60637, USA; Department of Oncology, Qingdao Cancer Institute, The Affiliated Hospital of Qingdao University, Qingdao University, Qingdao 266071, China; Ben May Department for Cancer Research, The University of Chicago, Chicago, IL 60637, USA

Metabolism and metabolic enzymes are differentially regulated in normal and tumor cells, resulting in specific metabolic features of cancer cells to support their rapid proliferation and migration, and counteract metabolic and genotoxic stress during cancer progression (Wang et al., 2018). These metabolic features include the Warburg effect, which is reflected by high rates of glycolysis with elevated levels of lactic acid regardless of oxygen levels, a substantial increase of anabolism, and reprogrammed catabolism and redox homeostasis (Li et al., 2018; Wang et al., 2018). Interestingly, metabolic enzymes are found to have moonlighting functions that use proteins as substrate and therefore regulate diverse cellular functions. In this paper, we define metabolic enzymes as a group of enzymes that catalyze conversion of metabolites during energy homeostasis, and we highlight that metabolic enzymes can not only function as protein kinases, but also act as protein phosphatases.

One of the key advances in understanding the multifaceted roles of metabolic enzymes is the demonstration of their kinase activity for proteins, in addition to its conventional activity for metabolites (Lu and Hunter, 2018). Pyruvate kinase M2 (PKM2), phosphoglycerate kinase 1 (PGK1), phosphoenolpyruvate carboxykinase 1 (PCK1), ketohexokinase isoform A (KHK-A), hexokinase (HK)2, choline kinase α2, 6-phosphofructo-2-kinase (PFKFB3), and nucleoside diphosphate kinase 1 and 2 (NME1/2) were revealed as protein kinases that can phosphorylate protein substrates, thereby modulating diverse cellular functions, such as gene expression, cell cycle progression, *de novo* nucleotide and lipid synthesis, lipid droplet hydrolysis, autophagy, and tumor immune evasion (Lu, 2012; Dasgupta et al., 2018; Lu and Hunter, 2018; Xu et al., 2020; Liu et al., 2021; Guo et al., 2022). Nevertheless, protein phosphorylation is controlled by two groups of enzymes with opposite activities, kinases and phosphatases (Lu and Hunter, 2009). It is therefore intriguing to ask if metabolic enzymes also possess noncanonical activities that functions as protein phosphatases.

A recent study by Wang et al. provided a provocative finding that gluconeogenic enzyme fructose-1,6-bisphosphatase 1 (FBP1) acts as a protein phosphatase to dephosphorylate histone H3 and suppress gene expression (Wang et al., 2022). As a metabolic enzyme, FBP1 catalyzes dephosphorylation reaction to hydrolyze fructose-1,6-bisphosphate (F1,6BP) to fructose 6-phosphate (F6P) in the cytosol. In normal human hepatocytes under glucose deprivation condition, FBP1 translocates into the nucleus in a protein kinase RNA-like endoplasmic reticulum kinase (PERK) activity-dependent manner (Fig. 1). PERK phosphorylates FBP1 at S170, leading to disassembling of FBP1 tetramer into the monomer and exposing the nuclear localization signal (NLS) in amino acids 17–30, which is otherwise largely buried in the interface of each dimer. The exposed NLS binds to importin α3 and then FBP1 gets translocated into the nucleus. The NLS of FBP1 is critical for this translocation, as R23A/K24A mutation abrogated nuclear translocation (Wang et al., 2022).

**Figure 1.**
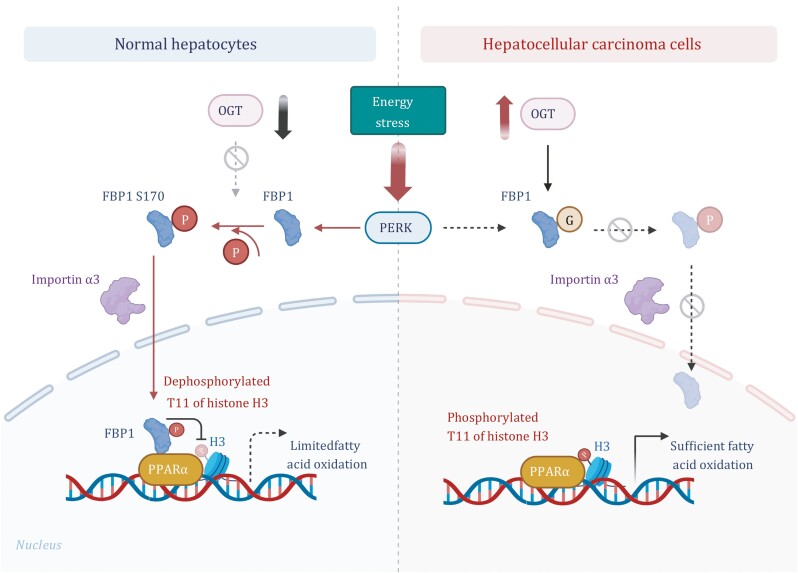
**A schematic depicting FBP1-regulated histone H3 dephosphorylation in normal and cancer cells.** In normal hepatocytes, glucose deprivation induces PERK-depended FBP1 S170 phosphorylation and translocation into the nucleus, where FBP1 binds to PPARα, dephosphorylates histone H3 at T11, and suppresses PPARα-mediated β-oxidation. In HCC cells, OGT mediated O-GlcNAcylation of FBP1 at S124 blocks PERK-depended FBP1 S170 phosphorylation and nuclear translocation of FBP1, promoting PPARα activity, β-oxidation, and tumor cell proliferation and survival.

In the nucleus, FBP1 interacted with PPARα, a master regulator of lipolysis and fatty acid β-oxidation gene expression. Expression of different FBP1 truncation mutants revealed that FBP1 Δ112–142 was unable to associate with PPARα, and mutations of the hydrophobic residues in this region showed that FBP1 V131 is the binding residue to PPARα. Chromatin immunoprecipitation-sequencing analyses showed that both FBP1 and PPARα were enriched at transcription start site regions and bound to a single dominant *de novo* motif of the genes related to mitochondrial and peroxisomal β-oxidation in response to glucose deprivation. Inhibition of nuclear translocation of FBP1 or its interaction with PPARα enhanced the transcriptional activity of PPARα and its downstream β-oxidation gene expression and subsequent fatty acid oxidation in mitochondria. These results indicate that FBP1 binds to PPARα and inhibits PPARα-mediated gene transcription (Wang et al., 2022).

To identify the mechanism underlying FBP1-repressed gene transcription, the authors demonstrated that PERK-phosphorylated FBP1 bound to purified histone H3. Notably, PERK1-phosphorylated FBP1 alters its catalytic domain conformation to enable close proximity of FBP1 C129 to phosphorylated histone H3 T11, as demonstrated by molecular dynamic simulation analyses. Importantly, FBP1 dephosphorylated phosphorylated T11 of purified histone H3 *in vitro* independent of the metabolic activity of FBP1. In addition, it was shown that C129 of FBP1 is in a reduced state and forms a covalent phosphoryl–cysteine intermediate in the process of H3 pT11 dephosphorylation. Mutation of C129 only abolished FBP1 protein phosphatase activity without affecting its metabolic activity, revealing the distinct catalytic features of FBP1 towards the protein and metabolite substrates with altered catalytic domain structures. Upon glucose deprivation, FBP1 dephosphorylated phosphorylated H3 at T11 in PPARα-regulated gene promoter regions in normal hepatocytes (Wang et al., 2022). Although molecular dynamic simulation and docking analyses with subsequent mutagenesis highlight the distinct catalytic features of FBP1 towards the protein and metabolite substrates, the exact protein structures, especially the catalytic domain structures, of FBP1 in both states will be necessary to fully reveal the catalytic mechanisms and related structural basis underlying the selective dephosphorylation of histone H3 pT11 by FBP1.

In contrast to normal hepatocytes, hepatocellular carcinoma (HCC) cells exhibited greatly reduced nuclear translocation of FBP1 upon glucose deprivation. Mass spectrometry analyses showed that FBP1 bound to O-linked *N*-acetylglucosamine (GlcNAc) transferase (OGT), which is frequently overexpressed in many types of cancer including HCC tissues (Wang et al., 2022). Notably, OGT mediated O-GlcNAcylation of FBP1 at S124 *in vitro* and *in vivo*, resulting in the disruption of FBP1–PERK1 interaction, PERK1-mediated FBP1 S170 phosphorylation, and nuclear translocation of FBP1. Consequently, in response to glucose deprivation, HCC cells exhibited substantially increased PPARα-regulated gene expression and β-oxidation levels and reduced apoptosis compared to normal hepatocytes. Animal studies showed that expression of O-GlcNAcylation-mutant FBP1 reduced tumor growth with correspondingly enhanced cell apoptosis rates, and these effects were eliminated by mutations of PERK-phosphorylated S170 or protein phosphatase activity-required C129. Analyses of human HCC specimens revealed lower levels of PERK-mediated FBP1 S170 phosphorylation and nuclear accumulation in the tumor specimens than in their adjacent normal tissues. In addition, FBP1 S170 phosphorylation levels were inversely correlated with OGT and β-oxidation enzyme levels and were positively associated with the survival time of HCC patients (Wang et al., 2022).

Human genome possesses a lot more metabolic enzymes (1653) than protein kinases and phosphatases (less than 800) (Romero et al., 2005). Upon metabolic stresses or oncogenic signals, cells reprogram the metabolic functions of metabolic enzymes, some of which become futile under these conditions, and confer novel distinct activities to these metabolic enzymes to compensate the cellular needs for cellular homeostasis, survival, and unlimited proliferations that could not be fully substantiated by protein kinases and phosphatases. The kinases for metabolites have been demonstrated to catalyze phosphorylation reaction for proteins that in turn regulates functions of their protein substrates (Yang et al., 2011, 2012a, 2012b; Yang and Lu, 2013; Alvarez et al., 2021; Fukushi et al., 2022). The demonstration of histone H3 dephosphorylation by gluconeogenic enzyme FBP1 (Fig. 1) suggests a new possibility for a metabolite phosphatase to function as a protein phosphatase (Gerber and Kettenbach, 2022). These findings indicate that cells can acquire a unique capability to regulate metabolic enzymes, not only in their canonical metabolic functions to meet their anabolic and catabolic needs, but also in their non-canonical functions as protein enzymes. Thus, the discovery of FBP1 as a protein phosphatase not only expands our current understanding of the functions of metabolic enzymes, but also suggests a possibility of noncanonical enzymatic activities of other metabolite phosphatases.

## Data Availability

The materials for the current study are available from the corresponding authors upon reasonable request.
